# Risk assessment of firefighting job using hybrid SWARA-ARAS methods in fuzzy environment

**DOI:** 10.1016/j.heliyon.2023.e22230

**Published:** 2023-11-11

**Authors:** Edris Soltani, Mostafa Mirzaei Aliabadi

**Affiliations:** a Student Research Committee, School of Public Health, Hamadan University of Medical Sciences, Hamadan, Iran; b Center of Excellence for Occupational Health, Occupational Health and Safety Research Center, School of Public Health, Hamadan University of Medical Sciences, Hamadan, Iran

**Keywords:** Risk assessment, Firefighters, SWARA method, ARAS method, Fuzzy sets

## Abstract

Identification and risk assessment of hazards using classic methods have various flaws, such as ambiguity and uncertainty in the data, inability to correctly reflect the human thinking style, failure to assign weight to criteria, use of predetermined data and tables, and the evaluator's role in the results of the risk assessment process. Therefore, developing these methods and creating approaches with higher accuracy and sensitivity is necessary. This study utilized a developed method that integrated SWARA's weighting methods and ARAS prioritization using Fine-Kinney criteria in a fuzzy environment to evaluate the risks of the firefighting job. The sensitivity analysis results confirmed the accuracy and applicability of the proposed method. Based on the study's results, the inhalation of harmful gases and smoke (R3) was identified as the most critical risk, while concern about the optimal operation (R21) was considered the least dangerous risk in firefighting.

## 1 Introduction

Firefighting is a hazardous occupation that puts firefighters at risk. They are exposed to increasingly complex psychological, physical, chemical, and biological hazards while performing duties such as extinguishing fires, rescuing those in need, and providing relief [1,2]. According to the National Fire Protection Association (NFPA), there are over 1.2 million firefighters in the United States. In 2021, there were 70 on-duty deaths among firemen in the US, which was a 13 % rise from the previous year [3]. According to estimates, there were 64,875 injuries to firefighters in 2020, a seven percent increase over the total number of injuries in 2018 and 2019 [4]. Besides the above injuries, there were 20,900 documented contacts with infectious diseases and 17,050 contacts with dangerous conditions during that year [4].

Based on the aforementioned cases, there is a need for risk assessment and the implementation of systematic, professional, and consistent measures to prevent accidents and manage occupational diseases faced by firefighters.

Risk assessment involves evaluating the risks associated with a hazard, assessing the effectiveness of existing controls, and determining the acceptability of the risks [5].

Using risk identification and risk assessment approaches, it is now possible to identify crucial sites and potential causes of accidents prior to an accident and adopt preventive and control actions. Risk assessment approaches can predict the likelihood and severity of events before they occur, identify which people are at risk and predict the level of potential harm they may face, and improve the quality of work life [[6], [7], [8]].

Based on the circumstances, quantitative or qualitative risk assessment methodologies such as the Fine-Kinney, ETA, FTA, FMEA, PHA, or HAZOP can be chosen and implemented [9].

However, Classical risk assessment methods, such as the classic Fine-Kinney method, have significant limitations. For example, they fail to evaluate the weighting of criteria such as consequence (C), probability (P), and exposure (E).

Another disadvantage of the classic Fine-Kinney technique is that same risk score may be obtained for two distinct risks.

As a result and considering that it is not possible to eliminate and reduce all risks due to financial constraints especially in low-income countries, approaches for more accurate assessment of occupational risks are required. Furthermore, the classic Fine-Kinney method traditionally quantifies people's opinions using predetermined criteria and values that do not fully reflect the human thinking style [10]. Consequently, using the classic Fine-Kinney approach increases ambiguity and uncertainty. Additionally, in the traditional Fine-Kinney method, there is no consideration of interaction between criteria [11].

In this study, fuzzy sets have been used to clarify ambiguities in human judgment and evaluations during the decision-making process. Furthermore, in order to determine the values of C, P, and E, linguistic terms were used instead of predefined data in classical methods. This approach aids in the reduction of uncertainty. Based on our knowledge and investigation, no study has been conducted so far to address the shortcomings of classical risk assessment methods by combining the SWARA weighting method and the ARAS prioritization method in the field of firefighting. Therefore, to address this gap, a combination approach based on these methodologies was proposed.

## 2 Literature review

Several studies have been conducted to address the shortcomings of the classic Fine-Kinney risk assessment approach.

Zhang et al. developed a risk assessment technique that combines the Fuzzy Analytical Hierarchy Process (FAHP) and Fine-Kinney methodologies to prioritize and categorize hazards in airport operating procedures. By utilizing such methods, risks can be prioritized, categorized, evaluated, and solutions can be proposed to prevent the occurrence of risks [12]. Gul et al. utilized the FAHP and the Fuzzy-Vlse Kriterijumsk Optimizacija Kompromisno Resenje (FVIKOR) approaches to overcome the limitations of the classic Fine-Kinney method. The weight of three significant risk assessment criteria was evaluated in their study [13].

Wang et al. used triangular fuzzy numbers, the MULTIMOORA method, and the Choquet integral approaches to overcome the limitations of the classic Fine-Kinney risk analysis [14]. Gul et al. presented a new occupational health and safety risk assessment strategy in rail transportation by combining the Fine-Kinney method with a fuzzy rule-based expert system to eliminate ambiguity in the classic Fine-Kinney method [5].

Derse et al. developed a model for measuring natural catastrophe risk using the Fine-Kinney approach. After calculating the risk score and identifying high-risk locations during a crisis, this study employed the Analytical Hierarchy Process (AHP)-based electrification method [15].

Gul et al. developed a risk assessment approach for identifying risks during wind turbine building and operation using the Fine-Kinney method in a fuzzy environment. The FAHP was used to weight the parameters in the Fine-Kinney, and then the fuzzy-VIKOR approach was used to prioritize the various risks associated with this activity [16].

In Yilmaz et al.'s study, the risks of lifting equipment used in building construction were rated using the Fine-Kinney approach. The causes of accidents were divided into seven primary categories based on prior occurrence records, and the weighted scores of these criteria were determined using the AHP method. The AHP weighted ratings were then multiplied by the Fine-Kinney scores, and the resulting values were used to prioritize specific hazards [17].

Tang et al.'s study presented a new risk assessment method for the risks of maintaining ballast tanks. They proposed a combined risk prioritization approach for Fine-Kinney by combining the TODIM and Fuzzy Best-Worst Method (FBWM) methods along with fuzzy sets. The results revealed the high accuracy and sensitivity of the suggested method [18].

Cebi et al.'s study used a combination of the Fine-Kinney method and a decomposed fuzzy set to assess the risk of welding arc hazards. Their study aimed to mitigate the uncertainty inherent in classical risk assessment methods. The results of their article revealed that harmful radiation and gas exposure pose a higher risk compared to electric shock, fire and explosion, and musculoskeletal disorders [19].

Fang et al.'s study utilized the critic and GLDS (gained and lost dominance score) methods to assess the risk of construction and defect rectification operations, as opposed to the classical Fine-Kinney method. Their study results demonstrated that the use of these methods in conjunction with fuzzy sets is capable of ranking potential hazards [20].

Tatar et al.'s conducted a research study with the aim of assessing the risk of work-related musculoskeletal disorders (WMSDs) in agriculture workers. They employed a combination of the Fine-Kinney and spherical fuzzy AHP-TOPSIS methods instead of the classical risk assessment approach for a more precise evaluation of the risk. The results of their study indicated the high accuracy and reliability of the proposed method [21].

Based on the review of the above literature, the Fine-Kinney approach is widely used in occupational safety risk assessment.

## 3 Material and methods

**Fig. 1:**
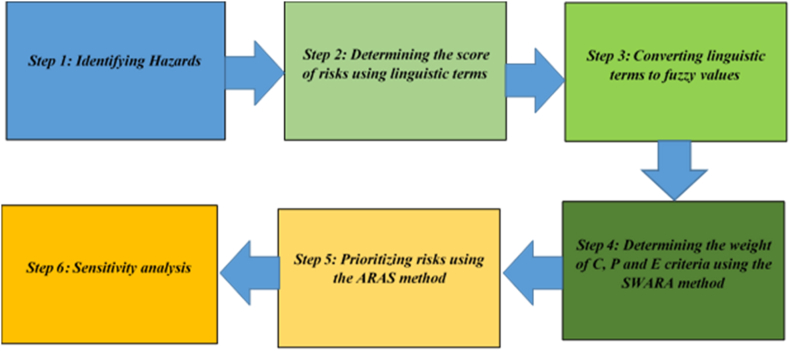
The proposed methodology.

Instead of the classical Fine-Kinney method, this section suggests a novel risk assessment model that combines a data-driven weighting system and the ARAS model (Fig. 1). The hazards related to the firefighting profession were first identified through the analysis of sources and expert interviews, which resulted in the identification of 25 important risks. Considering the limitations of the classical Fine-Kinney method, the risk scores for these risks were calculated in the second stage utilizing language terms provided by three experts and a fuzzy set. In the third stage, the weights of criterion C, P, and E were determined using the SWARA method after defuzzification. These numbers were then used to prioritize risks using the ARAS approach.

### 3.1 Fuzzy sets

**Fig. 2:**
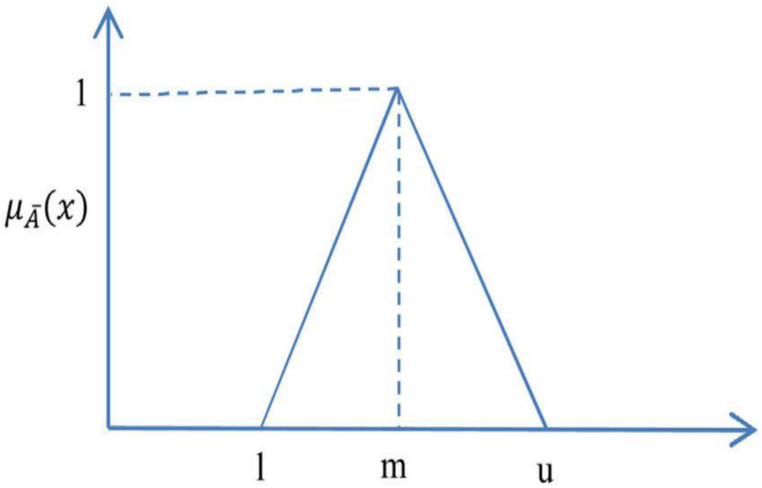
Triangular fuzzy number.

Lotfi Zadeh's fuzzy logic, which was developed in 1965, is a valuable technique for addressing the ambiguity and uncertainty inherent in human judgments and evaluations during decision-making [22,23]. Because of poorly specified goals and limits, many real-world decision issues involving people lack precision [5,22,24]. Linguistic terms provide a solution to these challenges [25]. When there is uncertainty, linguistic concepts can be converted into fuzzy numbers to reflect the opinions and experiences of decision-makers who possess sufficient knowledge and expertise in the relevant domain [26]. A triangle fuzzy number (TFN) is denoted by the triple F = (L, M, and U), where L, M, and U represent low, medium, and high fuzzy values, respectively. Fig. 2 illustrates a graphical representation of a triangular fuzzy number:

A triangular fuzzy number's membership degree is represented as follows [5].

(1) $$\mu \bar{A}=\left\{ \begin{array}{c} |0|x<l\\ |x-l/\left(m-l\right)|l\leq x\leq m\\ |\left(u-x\right)/\left(u-m\right)|m\leq x\leq u\\ |0|x\geq u \end{array}\right.$$

Due to the simplicity of performing mathematical operations on TFN, they exhibit high processing efficiency. Mathematical operations on fuzzy numbers such as ${\bar{A}}_{1}$ and ${\bar{A}}_{2}$ are as simple as the following:

The addition operation:

(2) $${\bar{A}}_{1}+{\bar{A}}_{2}=\left(l_{1}+l_{2}\;m_{1}+m_{2}\;u_{1}+u_{2}\right)$$

The subtraction operation:

(3) $${\bar{A}}_{1}-{\bar{A}}_{2}=\left(l_{1}-u_{2}\;m_{1}-m_{2}\;u_{1}-l_{2}\right)$$

The multiplication operation:

(4) $${\tilde{A}}_{1}x{\bar{A}}_{2}=\left(l_{1}xl_{2}\;m_{1}xm_{2}\;u_{1}xu_{2}\right)$$

### 3.2 Fine-Kinney method

Kinney created the Fine-Kinney technique in 1976 to estimate operational risks [27]. This technique determines the risk level using three criteria (consequence, exposure, and probability), and the risk priority number (RPN) is calculated by combining these elements [28]. Table 1 shows three parameter scales, while Table 2 indicates the interpretation of RPN.

**Table 1:** Scales of Fine–Kinney method.

Probability	value	Exposure	value	Consequently	value
“Might well be expected”	10	“Continuous”	10	“Catastrophe (many fatalities)”	100
“Quite possible”	6	“Frequent (daily)”	6	“Disaster (few fatalities)”	40
“Unusual but possible”	3	“Occasional (weekly)”	3	“Very serious (fatality)”	15
“Only remotely possible”	1	“Unusual (monthly)”	2	“Serious (serious injury)”	7
“Conceivable but very unlikely”	0.5	“Rare (a few per year)”	1	“Important (disability)”	3
“Practically impossible”	0.2	“Very rare (yearly)”	0.5	“Noticeable (minor first aid accident)”	1
“Virtually impossible”	0.1

**Table 2:** The RPN definitions.

Fine–Kinney risk score	Fine–Kinney risk situation
>400	“Very high risk; consider discontinuing operation”
200–400	“High risk; immediate correction required”
70–200	“Substantial risk; correction needed”
20–70	“Possible risk; attention indicated”
20>	“Risk; perhaps acceptable”

### 3.3 ARAS method

In 2010, Zavadsakas et al. introduced the Additive Ratio Assessment) ARAS (method for ranking and prioritization [29]. This method is also useful for risk assessment and prioritization, and it is regarded as one of the greatest multi-criteria decision-making strategies for selecting the optimal alternative. In the ARAS method, the best option is determined by maximizing the distance from negative factors and minimizing the distance from positive factors [30]. The ARAS method's steps are listed below in chronological order:

Step 1 Using actual data or expert opinions, create an initial decision matrix:

(5) $$x_{ij}=\left[\begin{array}{ccc} x_{11} & \cdots & x_{1n}\\ \vdots & \ddots & \vdots \\ x_{m1} & \cdots & x_{mn} \end{array}\right]\text{where}\left(i=1.\;2\ldots .m\right)\;\text{and}\;\left(j=1\;.\;2\;.\ldots .n\right)$$

in this matrix, m represents the number of options and n represents the number of criteria.

Step 2 The initial values of the criteria are normalized using Eqs (6) and (7):

(6) $$\text{For}\;\text{positive}\;\text{indicators}{\bar{x}}_{ij}=\frac{x_{ij}}{\sum_{i=0}^{m}x_{ij}}$$

(7) $$\text{For}\;\text{negative}\;\text{indicators}x_{ij}=\frac{1}{x_{ij}^{*}};{\bar{x}}_{ij}=\frac{x_{ij}}{\sum_{i=0}^{m}x_{ij}}$$

In these equations: $x_{ij}$ = The characters of the decision matrix.

Step 3 the created normal decision matrix should be balanced. For this purpose, the weights calculated by the weight determination methods for each criterion are multiplied in all the regions under the same criterion. Eq. (8) is used to obtain the normal weight values for all criteria:

(8) $${\hat{x}}_{ij}={\bar{x}}_{ij}w_{j};i=\bar{0,m}$$

In this Eq, $w_{j}$ is the weight (importance) of criterion j and ${\bar{x}}_{ij}$ is the normalized rank of criterion j.

Step 4 Using the desirability function described by Eq. (9), the utility of each option is determined. The best option is the one with the highest utility. The optimality function of each option is denoted by $S_{i}$ and is calculated as follows:

(9) $$S_{i}=\sum_{j=1}^{n}{\hat{x}}_{ij};i=\bar{0,m}$$

where $S_{i}$ is the optimality function of option i.

Step 5 the utility degree is calculated using Eq (10):

(10) $$K_{i}=\frac{S_{i}}{S_{0}};i=\bar{0,m}$$

In this Eq, $S_{i}$ and $S_{0}$ indicate the values of the degree of desirability obtained from Eq. (9). It is evident that the calculated values of $K_{i}$ fall within the range of [0, 1], and they can be organized in ascending order to indicate the priority ranking of the desired problem.

### 3.4 SWARA method

The SWARA method is a multi-criteria decision-making approach for determining the weights of decision-making criteria and indicators. Crossline introduced it in 2010 [31]. In this method, the opinions of experts play a crucial role. Experts vote on each criterion, and insignificant items are eliminated. The steps of the SWARA method are listed below:

Step 1 First, the desired criteria are listed in order of their importance. Assigned to the highest categories, while the least important criteria are assigned to the lowest categories. The most significant criterion is given a score of one in this procedure.
Step 2 The relative importance of each criterion ($s_{j}$) is calculated by comparing its importance to the previous criterion.
Step 3 The coefficient $c_{j}$, which is a function of the relative importance value of each criterion, is calculated using Eq (11):

(11) $$c_{j}=s_{j}+1$$

Step 4 The modified weight value ($s_{j}^{\prime }$) is calculated using Eq (12):

For the most important criteria: $s_{j}=0$.

(12) $$s_{j}^{\prime }=\frac{s_{j-1}^{\prime }}{c_{j}}$$

Step 5 The final weight of the indicators ($w_{j}$), also known as the normalized weight, is determined using Eq (13) in the last step of the SWARA method:

(13) $$w_{j}=\frac{s_{j}^{\prime }}{\sum_{J=1}^{N}s_{j}^{\prime }}$$

## 4 Case study

The risk criteria, namely C, P, and E, were initially calculated using the classic Fine-Kinney approach, and a risk matrix was built using the qualitative language terms of the experts.

Using the values in Table 3, a fuzzy decision matrix was generated. Following the summarizing and fuzzifying of experts' opinions, these values were used to determine the weight of the C, P, and E criteria using the SWARA weighting system. Subsequently, various risks were ranked using the weighted values of the criteria and the ARAS method.

**Table 3:** Average triangular fuzzy numbers range of 7°.

Linguistic variable	Fuzzy equivalent
Very low (VL)	(0,0,1)
Low (L)	(0,1,3)
Low to moderate (LM)	(1,3,5)
Moderate (M)	(3,5,7)
Almost high (AH)	(5,7,9)
High (H)	(7,9,10)
Very High (VH)	(9,10,10)

This study focuses on firefighters who face a variety of challenges, such as mental and emotional stress, as well as physical and chemical injuries that can endanger their health. Identifying and assessing these risks is a critical step in decreasing financial and physical losses among professionals in this field. The following steps were then undertaken to prioritize the risks associated with the firefighting job and evaluate the effectiveness of the suggested method.

### 4.1 Compiling and extracting a list of firefighting risks

The first step involved compiling a comprehensive list of potential hazards from various sources, including a literature review and interviews with a panel of experts [[32], [33], [34], [35], [36]]. Table 4 shows the findings of identifying 25 important risks.

**Table 4:** Determination the risks of the firefighting job.

Risk code	Risk description
R1	Hot air and radiant heat exposure
R2	Contact of the body with hot surfaces
R3	Inhalation of harmful gases and smoke
R4	Alarm anxiety causes immediate anxiety
R5	cancer of the lungs.
R6	heart attacks
R7	Burn
R8	Injuries caused by falling objects
R9	Building collapse
R10	Improper body posture during duty
R11	Accidents with vehicles caused by haste
R12	Ionizing radiation exposure
R13	Explosion
R14	Transport and handling of heavy equipment
R15	Inadequate use of personal protective equipment
R16	Inadequate experience
R17	Tiredness, a lack of sleep, and shift work
R18	Injuries sustained during training, maneuvering, and training
R19	Traffic and driving stress
R20	Contact with electricity and electrocution
R21	Concern about optimal operation
R22	Vibration of the hands and arms
R23	Animal bites as a result of live feeding
R24	Getting stuck in pits and wells
R25	Reduced visibility due to darkness and smoke

### 4.2 Interviewing experts to determine the risk score

To provide an accurate response, the term “expert” must be properly defined. An expert participant in the risk assessment process was defined in this study as someone who met the following criteria: 1) familiarity with multi-criteria decision-making systems; 2) understanding of the Fine-Kinney technique; 3) knowledge of the firefighting profession; and 4) having a bachelor's degree or higher. Three specialists with the greatest expertise and knowledge in the subject were chosen to carry out the risk assessment process based on these criteria. Table 5 lists the characteristics of the experts who took part in this study.

**Table 5:** Characteristics of experts.

	Academic Degree	Job	Work Experience
Expert 1	Ph.D	Crisis Manager	17 years
Expert 2	M.S	Fire Chief	22 years
Expert 3	Ph.D	Occupational Health and Safety Officer	13 years

They were then asked to use qualitative linguistic terms to rate the C, P, and frequency of E for the risks associated with firefighting work (Table 6). Using these ratings as well as Eqs (1), (2), (3), (4), (5), an initial risk matrix was built. Following the collection of expert opinions and conversion of fuzzy numbers into crisp values, a numerical matrix was constructed and used in the next step (Table 7).

**Table 6:** Qualitative risk values determined by experts.

Risk	Expert 1			Expert 2			Expert 3		
	C	P	E	C	P	E	C	P	E
R1	VL	VH	H	VL	H	H	VL	AH	H
R2	M	H	H	L	H	M	VL	AH	M
R3	VH	H	H	H	H	M	H	H	M
R4	L	M	M	VL	H	M	VL	LM	M
R5	VH	VL	VL	VH	VL	VL	H	VL	VL
R6	VH	LM	L	VH	L	L	VH	M	M
R7	VH	VH	H	H	M	M	M	H	M
R8	H	M	H	H	M	L	AH	M	L
R9	VH	M	LM	H	M	L	H	L	L
R10	VL	H	H	VL	M	AH	VL	M	AH
R11	AH	LM	VL	VL	VL	VL	L	M	M
R12	L	M	M	VL	VL	L	M	L	L
R13	VH	VH	L	VH	M	VL	VH	M	L
R14	L	M	L	VL	L	L	L	L	L
R15	M	M	M	VL	H	M	AH	M	L
R16	LM	LM	M	VL	M	LM	L	M	M
R17	LM	LM	M	VL	L	L	L	L	L
R18	M	M	VL	L	VL	L	L	VL	L
R19	VL	VL	L	VL	L	LM	L	L	L
R20	VH	VH	L	H	L	VL	AH	M	M
R21	VL	VL	L	VL	VL	VL	VL	L	VL
R22	VL	VL	L	VL	VL	L	VL	VL	VL
R23	M	M	M	L	M	M	L	M	M
R24	M	M	H	AH	M	AH	M	M	AH
R25	M	M	H	AH	H	AH	M	M	AH

**Table 7:** Initial matrix.

Risk	C	P	E
R1	0.25	8.39	8.75
R2	0.68	8.11	6
R3	9.06	8.75	6
R4	0.36	5	5
R5	9.39	0.25	0.25
R6	9.75	2.4	1.84
R7	7.48	7.48	6
R8	8.11	5	2.16
R9	9.06	2.77	1.61
R10	0.25	6	7.53
R11	0.75	0.81	0.47
R12	0.68	0.68	1.84
R13	9.75	6.19	0.52
R14	0.52	1.84	1.25
R15	0.99	6	2.77
R16	0.61	4.18	4.18
R17	0.61	1.84	1.84
R18	1.84	0.25	0.52
R19	0.36	0.52	1.6
R20	8.39	3.26	0.68
R21	0.25	0.36	0.36
R22	0.25	0.25	0.52
R23	1.84	2.77	5
R24	5.75	5	7.53
R25	5.75	7.23	7.53

### 4.3 Sensitivity analysis

As a first validation study, the proposed method was compared to the VIKOR and Combined Compromise Solution (CoCoSo) methodologies to determine its applicability. The findings of this comparison are shown in Table 8. Based on the results, R3 was identified as the most critical risk in all three methods. By comparing the results obtained from the comparison of these methods, it was determined that the proposed method has a high potential for risk prioritization.

**Table 8:** Comparison of the proposed method with the VIKOR and COCOSO methods.

Risk	ARAS	VIKOR	CoCoSo
Priority number			
R3	1	1	1
R7	2	2	2
R25	3	3	4
R13	4	5	3
R24	5	6	5
R21	25	25	25
R22	24	24	24
R11	23	22	22
R19	22	23	23
R12	21	21	21

After validating the proposed model's reliable performance, sensitivity analysis was done in the second stage. Researchers will use sensitivity analysis to analyze the effects of changes in one variable on other variables. We performed a sensitivity analysis to examine the variation in risk ranking as a result of changes in criteria weights. Various weight vectors of the Fine-Kinney criteria were used.

A total of four combinations were generated. Table 9 shows the weight vectors of Fine-Kinney criteria selected for sensitivity analysis Table 10 displays the ranking orders for the sensitivity analysis. Table 10 shows that as the weight vector changes, the ranking orders of risks vary. As a result, our proposed approach is sensitive to the weights of the Fine Kinney risk criteria. The sensitivity analysis revealed that, even when the criteria weights were changed, R3 remained the most significant risk. Based on the results of the sensitivity analysis, it can be concluded that the ranking results obtained from our proposed method are reliable and applicable.

**Table 9:** The weight vectors created for the sensitivity analysis.

Weight vector	Parameter	Weight value
Weight vector-1 (W1)	Consequence	0.482
	Probability	0.301
	Exposure	0.215
Weight vector-2 (W2)	Consequence	0.550
	Probability	0.250
	Exposure	0.200
Weight vector-3 (W3)	Consequence	0.333
	Probability	0.333
	Exposure	0.333
Weight vector-4 (W4)	Consequence	0.200
	Probability	0.400
	Exposure	0.400

**Table 10:** Changes in risk rankings with variations in criteria weights.

Risk	Ranking order of the risks			
	W1	W2	W3	W4
R1	10	11	5	3
R2	12	12	7	6
R3	1	1	1	1
R4	16	16	13	10
R5	11	10	16	17
R6	7	5	10	13
R7	2	2	2	4
R8	6	6	8	9
R9	8	8	11	15
R10	13	13	9	7
R11	23	22	23	23
R12	21	21	20	20
R13	4	3	6	8
R14	19	20	19	19
R15	15	15	15	11
R16	17	17	17	14
R17	18	18	18	18
R18	20	19	22	22
R19	22	23	21	21
R20	9	9	12	16
R21	25	25	25	25
R22	24	24	24	24
R23	14	14	14	12
R24	5	7	4	5
R25	3	4	3	2

## 5 Results

To calculate the weights of the criteria, the data weighting system and the SWARA technique were initially utilized. Table 11 shows the results of weighting the criteria and the processes of the SWARA technique.

**Table 11:** Weights of C, P and E criteria.

Criteria	relative importance	$c_{j}$	initial weight	Normal weight
C	1	1	1	0.482
P	0.6	1.6	0.625	0.301
E	0.4	1.4	0.446	0.215

According to experts' opinions, criterion C was more important than criteria P and E. Furthermore, criterion P was more significant than Criterion E. Criteria C, P, and E received values of 1, 0.6, and 0.4, respectively.

Using this method, the weight for the C criterion was determined to be 0.482, while the weights for the P and the E criteria were 0.301 and 0.215, respectively.

The ARAS technique was utilized in the second stage to evaluate and prioritize risks associated with the firefighting profession.

The values $S_{i}$ of and $K_{i}$ were calculated, and the prioritizing of recognized risks in the firefighting profession was decided based on these values. Table 12 displays these values.

**Table 12:** The final weight of options and their priority.

Risk	$S_{i}$	$K_{i}$	Priority
R1	0.051	0.501	10
R2	0.045	0.443	12
R3	0.091	0.893	1
R4	0.031	0.304	16
R5	0.050	0.496	11
R6	0.063	0.623	7
R7	0.078	0.773	2
R8	0.064	0.628	6
R9	0.060	0.593	8
R10	0.040	0.395	13
R11	0.008	0.076	23
R12	0.011	0.104	21
R13	0.072	0.707	4
R14	0.012	0.116	19
R15	0.031	0.310	15
R16	0.027	0.270	17
R17	0.014	0.136	18
R18	0.012	0.116	20
R19	0.008	0.076	22
R20	0.056	0.550	9
R21	0.003	0.033	25
R22	0.003	0.034	24
R23	0.031	0.311	14
R24	0.065	0.646	5
R25	0.073	0.716	3

## 6 Discussion

By using qualitative and quantitative risk assessment techniques, it is possible to determine the individuals at risk and the potential severity of harm they may experience [37].

Classical risk assessment methods have several areas that could be improved, including the same RPN for distinct risks, the inability to assign weights to different criteria, uncertainty, and ambiguity. The SWARA weighting method was used to determine the weight of the criteria. The SWARA method community includes experts in the field of study.

In this method, the purposeful sampling technique is employed, and experts play an important role in evaluating the calculated weights. Expert views are used to rank the desired criteria in order of priority. The more important criteria are assigned to higher categories, while the criteria that are less important are assigned to lower categories. The relative importance of each criterion to the last criteria is then determined.

Additionally, each expert makes a substantial contribution to determining the value of each criterion based on their implicit knowledge, information, and experiences. Subsequently, by averaging the group ranks provided by the experts, the weight of each criterion is determined. In this strategy, experts must gather and give their views as a group. During the process of evaluating the criteria, the researcher should take detailed notes and summarize the experts' opinions in order to determine their relative weights.

Next, the coefficient $\left(c_{j}\right)$, which is a function of each criterion's relative importance value, is calculated using Eq (11). In the fourth step, the initial weights of the criteria are calculated using Eq (12). In this step, the weight of the first and most important criterion is set to 1.

The final weights of the indicators, also known as the normalized weights, are determined using Eq (13) in the final phase of the SWARA technique. Based on the experts opinions, the C was identified as the most important selection criterion, and the P and E criteria's relative importance was 0.6 and 0.4, respectively, compared to the C criterion.

According to expert opinion, the C criterion is the most important criterion for assessing the risks of firefighting. After calculating the coefficient and the initial weights, the normalized final weights were determined as follows: the C criterion with a weight of 0.482, the P criterion with a weight of 0.301, and the E criterion with a weight of 0.215.

The SWARA method weights were used in the following steps to prioritize and determine the most severe risks. By incorporating weight assignment into the criteria impacting the risks, one of the main limitations of classical risk assessment methods was removed.

The ARAS approach was then used to prioritize various risks in the firefighting job. This method helps identify the most significant risks in this field. To prioritize the risks using the ARAS technique, a scoring matrix of indicators based on the criteria was built. The dimensionless decision-making matrix was created in the second stage using the linear method and Eq (6) for positive criteria and Eq (7) for negative criteria.

In the third phase, the dimensionless matrix was converted into a balanced dimensionless matrix using Eq (8). In the weighted dimensionless matrix, the weights of the criteria, which were calculated using the SWARA technique, were multiplied by the dimensionless matrix. This final matrix is known as the dimensionless weighted matrix. Finally, the optimality function ($S_{i}$) was calculated using Eq (9) to assess the prioritization of the risks. The sum of the values in the matrix is one. The option with the highest, $\left(S_{i}\right)$ value is considered the best. Using Eq (10), the utility degree ($K_{i}$) is calculated, allowing for the prioritization of various risks in the firefighting job.

Based on this, the values of ($K_{i}$) for inhalation of harmful gases and smoke (R3), Burns (R7), and reduced visibility due to darkness and smoke (R25) were 0.893, 0.733, and 0.716, respectively. As a result, the proposed model identifies these three risks as the most significant in the firefighting profession. Furthermore, rushing-related vehicle accidents (R11), hand and arm tremors (R22), and concerns about optimal performance (R21) had lower importance in this profession, with ($K_{i}$) values of 0.076, 0.034, and 0.033, respectively.

To evaluate the applicability of the current study, a comparison was done between the proposed method and two frequently used MCDM methods in risk assessment: VIKOR and CoCoSo [13,38]. The findings of the sensitivity analysis confirmed that the most significant risk in firefighting is the inhalation of harmful gases and smoke (R3).

Furthermore, the rankings of the three highest-level risks, R3, R7, and R13, were consistent across all three methods. Additionally, the ranking of the five least important risks was the same in the proposed method and the comparative method. Minor differences between the suggested method and the other two methods were detected, owing to the close values of specific risks. For example, R13 risk was ranked fourth in the ARAS method and fifth in the VIKOR method. Additionally, risk R24 is ranked fifth in the ARAS and CoCoSo methods and sixth in the VIKOR approach.

Overall, the comparison and prioritization using different MCDM methods demonstrated that the proposed method in this study has acceptable validity and accuracy. Table 8 displays the results of the three prioritization methods, namely ARAS, VIKOR, and CoCoSo, for ten high- and low-priority risks. In addition, the results of the sensitivity analysis confirmed the credibility and applicability of the proposed model.

## 7 Conclusion

To analyze risks in the firefighting job, this study suggested an integrated decision-making model based on the SWARA weighing system and the ARAS prioritization technique. According to the current study, the most serious risk identified was the inhalation of harmful gases and smoke (R3), whereas the least serious risk was concern about optimal performance (R21). Additional measures could be considered in future investigations. These measures may include revising the examined criteria to make them more comprehensive, increasing the number of experts involved in the risk assessment process, testing the proposed method in other industries to ensure its applicability, accuracy, and precision, and comparing it with the findings of the present study.

## Ethics statement

This paper was supported by the vice president for research at 10.13039/501100004697Hamadan University of Medical Sciences (Ethics Committee No: IR. UMSHA.REC.1402.075, Grant No: 140203021585). Also the experts present in the study were fully aware of the work process and expressed their consent to participate in the study in writing.

## Data availability statement

Data included in article/supp. Material/referenced in article.

## CRediT authorship contribution statement

**Edris Soltani:** Writing – review & editing, Writing – original draft, Formal analysis, Data curation, Conceptualization. **Mostafa Mirzaei Aliabadi:** Writing – review & editing, Visualization, Validation, Supervision, Software, Resources, Project administration, Methodology, Investigation, Funding acquisition.

## Declaration of competing interest

The authors declare that they have no known competing financial interests or personal relationships that could have appeared to influence the work reported in this paper.
